# The concept of heart team in cardiac diseases: the patient is back as a priority in medical decisions*

**DOI:** 10.1590/S1516-31802012000400002

**Published:** 2012-09-11

**Authors:** Walter José Gomes, Domingo Marcolino Braile

**Affiliations:** I MD, PhD. Associate Professor of Cardiovascular Surgery, Escola Paulista de Medicina, Universidade Federal de São Paulo, São Paulo, Brazil.; II MD, PhD. Emeritus Professor and Pro-Rector of Faculdade de Medicina de São José do Rio Preto (Famerp); Full Professor at Universidade Estadual de Campinas (Unicamp), Campinas, São Paulo, Brazil.

The patient is back at the center of attention and should be the priority in medical decision-making. This is the recommendation of the recent guidelines on coronary revascularization from the ESC/EACTS (European Society of Cardiology/European Association for Cardio-Thoracic Surgery) and the ACC/AHA (American College of Cardiology/American Heart Association).^1^^,^^2^^,^^3^

Through compilation and synthesis of the best scientific evidence for decision-making relating to patients with coronary artery disease, these guidelines reinforce and specifically recommend adoption of a multidisciplinary approach in clinical decision-making, involving discussion between cardiologists, interventional cardiologists and the cardiac surgeons, in order to provide the best patient-oriented care. The decision-making process and provision of medical information to patients should be guided by the approach known as the “four principles” for health ethics: autonomy, beneficence, non-maleficence and fairness.^1^

Establishment of a multidisciplinary team has the aim of enabling balanced clinical decision-making through evidence-based protocols that are designed in collaboration between cardiologists, interventional cardiologists and cardiac surgeons. The cases of all patients with stable coronary artery disease must be discussed by a multidisciplinary heart team before making decisions on the type of revascularization procedure to be indicated or deciding to keep the patients on medication treatment only.

With the mounting complexity of cases and severity of patients’ conditions, there is often a need to involve other experts, depending on the comorbidities that are observed, such as diabetes specialists, general practitioners, nephrologists, intensivists, referral physicians or anesthesiologists, such that their combined experience and expertise can decisively contribute towards the most appropriate decision. Therefore, it is essential for the heart team to review each patient’s case before issuing a treatment recommendation relating to that patient’s heart condition. Hospitals and healthcare institutions are encouraged to establish multidisciplinary teams so that procedures can be guided towards what is most appropriate and necessary for patients. One study in the United States has suggested that 50% of patients may not have received adequate treatment, in the way proposed through guidelines.^4^ Consequently, the Centers for Medicare and Medicaid Services (CMS) is implementing a program in which Medicare auditors will review all medical records and assess the adequacy of the procedures and devices used and the procedure applications or hospital admissions made, prior to paying those bills. The new program went into effect in January 2012.^5^

Correct indications for procedures not only result in benefits for patients but also result in reduced costs for public and private payers, which can serve to redirect resources to areas that are most in need. It has been suggested that standard institutional protocols compatible with the current guidelines can be used to avoid the need for systematic case-by-case review of all diagnostic angiograms. This concept essentially gives shape to the collaboration among specialties that is necessary in order to match their skills and expertise through complementary approaches.

However, the concept goes beyond this: patients should also have active participation in the therapeutic decision-making process. Information provided to patients needs to be objective, impartial and based on current scientific evidence, and must be understandable, accessible and consistent. Informed consent requires transparency, especially in situations in which no consensus exists in relation to the treatment indicated, such as in cases of percutaneous intervention or other surgical or medication.

Patients need to understand the risks, benefits and uncertainties associated with their disease and its treatment. These measures are mandatory, and incomprehensible technical language should be avoided. The emphasis needs to be placed on use of consistent terminology that is understandable to patients. The information provided and medical decisions made should take into consideration the benefits relating to the procedure, the short-term risks and the expected long-term outcomes and risks. Risks and benefits in terms of survival, relief of angina, quality of life and the potential need for late reintervention should be clearly informed. It is also important to note that all other interests in decision-making, among the professionals involved in the different treatment options, should be clearly made known to patients. It is recommended that patients should be given enough time (even several days, if necessary) between diagnostic catheterization and intervention, so that they can reflect on the diagnostic angiographic findings, seek a second opinion if desired, or discuss the results and consequences with the cardiologist or referring physician.

The growing public demand for transparency in relation to implementation of procedures and hospital outcomes gives rise to a need to avoid anonymous treatment. Patients have the right to know who is about to treat them and to have information about the level of operator expertise and volume of procedures at the hospital where the treatment will take place. In addition, they need to be assured that all the treatment options are available and that on-site surgery can be offered, should the need arise.

The concept of the heart team is expanding and must necessarily also be applied to new technologies that come onto the market, with a multidisciplinary approach towards selection and treatment of patients with valve disease, use of transcatheter prostheses and interventions relating to atrial fibrillation.^6^

Therefore, through reestablishment of an obvious concept that until now had been forgotten and relegated, the multidisciplinary approach towards heart disease represents a new effort that, with the support of physicians, hospitals and specialty societies, will again restore patients as the primary goal of care and medication treatment, and the greatest beneficiaries from decision-making, taken as a whole.
